# Toughening Mechanism of Directional Fabric Woven Net and/or Non-Directional Short-Cut Fiber-Reinforced Sprayed Cement Mortar Thin-Plates

**DOI:** 10.3390/ma16124418

**Published:** 2023-06-15

**Authors:** Jiyang Wang, Dan Yu, Fujiang Mu, Xiaohua Ji, Yu Peng

**Affiliations:** 1 Institute of Engineering Mechanics, China Earthquake Administration, Harbin 150080, China; 2 Center for Balance Architecture, Zhejiang University, Hangzhou 310007, China; 3 College of Civil Engineering and Architecture, Zhejiang University, Hangzhou 310058, China; 4 The Architectural Design & Research Institute of Zhejiang University Co., Ltd., Hangzhou 310007, China; 5 China Construction Eighth Engineering Division Co., Ltd., Shanghai 200112, China

**Keywords:** toughening mechanism, fabric woven net, shot-cut fiber, cement mortar, thin plate

## Abstract

In this paper, a non-directional short-cut polyvinyl alcohol fiber (PVA), directional carbon-glass fabric woven net, and a combination of these fibers were used to reinforce sprayed cement mortar (named FRCM-SP, FRCM-CN, and FRCM-PN accordingly), and the direct tensile and four-point bending tests of these three types of thin plates were conducted. It was shown that the direct tensile strength of FRCM-PN reached 7.22 MPa under the same cement mortar matrix, which was 175.6% and 198.3% higher than that of FRCM-SP and FRCM-CN, respectively; the ultimate tensile strain of FRCM-PN was 3.34%, which was 65.3% and 1291.7% higher than that of FRCM-SP and FRCM-CN, respectively. Similarly, the ultimate flexural strength of FRCM-PN reached 33.67 MPa, which was 182.5% and 519.6% higher than that of FRCM-SP and FRCM-CN, respectively. In addition, the tensile, bending toughness index, and residual strength factor of FRCM-PN were much higher than those of FRCM-SP and FRCM-CN, indicating that the incorporation of non-directional short-cut PVA fibers improved the interfacial bonding properties between the cement mortar matrix and the fiber yarn and significantly enhanced the toughness and energy dissipation capacity of the sprayed cement mortar. Therefore, the use of a certain amount of non-directional short-cut PVA fibers can improve the interfacial bonding properties between the cement mortar and the fabric woven net while ensuring the spraying performance and significantly improving the reinforcing and toughening effect on the cement mortar to meet the demand for large-area rapid construction and structural seismic reinforcement.

## 1. Introduction

Concrete has been the most widely used building material since the introduction of Portland cement in the early 19th century. However, the quasi-brittle behavior of concrete materials may be an important reason for concrete cracking and limit the service life of concrete structures. In recent years, fabric-reinforced cement mortar (FRCM) has drawn extensive attention from researchers and engineers because of its advantages of being lightweight and having a higher load-carrying capacity, crack limitation, and corrosion resistance than ordinary concrete [1,2,3,4,5]. Moreover, FRCMs have started to be applied to improve the load-carrying capacity or durability of structure members [6,7,8,9].

By laying fiber yarns along the direction of tensile stress and designing a reasonable raw material ratio of cement mortar matrix, it can theoretically improve the utilization effect of fibers and the mechanical properties of FRCM effectively. However, the quasi-brittle characteristic of the cement mortar matrix may contribute to the poor interface property and poor collaboration work capacity between fiber yarns and the matrix. The investigation conducted by Evans et al. [1] and Häußler et al. [2] demonstrated that the interfacial bond property between fiber yarn and concrete is usually subjected to deficiencies because only the outer filaments of oval yarns, which consist of thousands of filaments, are fully embedded into the matrix where direct bond strength occurs, while central filaments bear tensile stress entirely or partially through friction between filaments. When cracks occur, the outer filaments may be peeled off the cement mortar or broken due to the stress concentration. As a result, the synergistic deformation between the fiber yarn and the cement mortar matrix may fail, making it difficult to effectively utilize the directional reinforcement effect of the fiber yarn.

For improving the mechanical performance and the fabric woven net utilization rate of FRCM, experimental tests and theoretical analyses have been carried out by national and international researchers. Reinhart et al. [10] conducted four-point bending tests to study the influences of fabric woven net type, impregnated epoxy resin, and the prestress on the mechanical performance of FRCM plates. The results show that the first-crack load, ultimate load, and cracking-control ability of FRCM plates could be improved by prestressing the impregnated fabric woven net. Zhu et al. [11] conducted experimental tests on the flexural performance of alkali-resistant (AR) glass fiber FRCM. The results show that the capacity of energy absorption could be increased by increasing the number of fabric-woven net layers. Du et al. [12] carried out four-point bending tests to study the effect of basalt fabric woven net layers, prestress levels, and the addition of short-cut steel fibers on the flexural performance of FRCM plates. The results showed that the maximum ultimate flexural strength of FRCM reinforced by five layers fabric woven net was 26.25 MPa and the maximum deflection was 10.788 mm; the ultimate flexural strength of FRCM reinforced by four layers fabric woven net and 1.6% volume ratio of short-cut steel fibers was 23.65 MPa, the maximum deflection was 12.570 mm, and the maximum toughness was 6.793 N·m. Their mechanical performance was much higher than those of the FRCM reinforced by a one-layer fabric woven net. Hinzen et al. [13] and Barhum et al. [14] conducted a series of tests on the mechanical performance of FRCM plates with short-cut glass fibers, carbon fibers, and hybrid fibers. Moreover, the effect of different fibers on the increase in ultimate strength was also studied. Yin et al. [15] improved the mechanical performance of FRCM by adhering sand on an epoxy-resin-impregnated fabric woven net and adding short-cut polypropylene fibers. However, regardless of the presence of short-cut fibers in the matrix, the matrix of FRCMs is still quasi-brittle, which limits its mechanical properties. Due to the small number of cracks and large crack spacing, the crack width still cannot meet the requirements of structural codes in extreme environments, but the multi-crack cracking behavior can make the crack width less than 0.05 mm, which can meet the general requirements of structural durability. In addition, the technology to cause the the carbon fiber fabrics to be impregnated or prestressed may be complex and inconvenient for engineering applications. The strength of FRCMs with a layer of fabric woven net is insufficient, as demonstrated in the literature [12], while the multiple layers of the fabric woven net may not be fully used, which limits the mechanical and economic efficiency of FRCMs. Therefore, it is necessary to improve the toughness of the FRCMs matrix to guarantee the interfacial bonding performance between the fabric-woven net and the matrix, and to be able to effectively utilize the strength of fabric-woven nets.

In recent decades, a kind of short-cut-fiber-reinforced cementitious composite with high ductility has been designed and developed based on the micromechanical principles first proposed by Li and Leung [16]. This kind of material, which is also referred to as engineered cementitious composites (ECCs) [17,18,19,20], strain hardening cementitious composites (SHCCs) [21,22,23], or ultra-high-toughness cementitious composites (UHTCCs) [24,25,26], exhibits significant pseudo strain hardening and multiple fine crack properties. the potential application of this material has been revealed by many researchers [27,28,29,30,31,32,33,34,35]. Moreover, for the failure criterion model of this kind of composite, Pang et al. [36] made two modifications to the Tsai-Wu micromechanics model. The addition of a single longitudinal tensile test to the modified expression reduces the average absolute error to 15.9%, which proves that the modified model can provide satisfactory failure predictions while greatly reducing the amount of testing required. In Kabir’s study [37], the presented checkerboard micromechanics model found by the employed Monte Carlo simulation exactly matches the experimental data and predicts the composite elastic constants more accurately than that found from other common methods, including the Halpin-Tsai theory. Li et al. [38] investigated the tensile and flexural properties of UHTCC and the ultimate strain capacity above 4% of UHTCC under uniaxial loading was demonstrated, and the width of saturated multiple cracks was controlled below 50 μm. In recent years, researchers began to apply UHTCC to replace the traditional FRCM cement mortar matrix, improving the toughness of the FRCM and the utilization of the fabric woven net. However, the conventional UHTCC has complex matrix components and high-volume admixture of polyvinyl alcohol (PVA) fibers in order to meet the characteristics of high strain hardening, resulting in high cost and poor flowability of the composite material to meet the requirements of large area thin layer construction. Kim et al. [39,40,41] developed a sprayable ECC based on the rheology-based design, which greatly benefits large-area construction due to its high construction efficiency. The experimental investigation on the mechanical properties and repair performance of this material demonstrated that sprayed ECC still maintains the pseudo strain hardening properties similar to conventional ECC, while the deformation capacity decreased by almost 50% when compared to conventional ECC. The investigation on sprayed UHTCC conducted by Xu et al. [42] also revealed a significant reduction when compared with conventional UHTCC, where the average ultimate tensile strength and strain of sprayed UHTCC is 2.3 MPa and 1.5%, respectively. Therefore, the ultimate tensile strength and strain of short-cut fiber-reinforced sprayed cementitious composites have the potential to be increased and can also work synergistically as a matrix material for FRCM together with fiber woven net to play a reinforcing and toughening role.

In this paper, uniaxial tensile and four-point bending tests were conducted on three types of FRCM thin plates added with non-directional short-cut PVA fibers, a directional carbon fiber woven net, and combined compound of the above two fibers with sprayed-type cement mortar as the matrix, and the ultimate tensile and flexural strength, toughness, and crack propagation of FRCM thin-plate were analyzed and discussed in order to obtain a new construction material with excellent mechanical properties, convenient construction process and low cost.

## 2. Experimental Program

### 2.1. Raw Materials and Mixed Proportion

**Figure 1:**
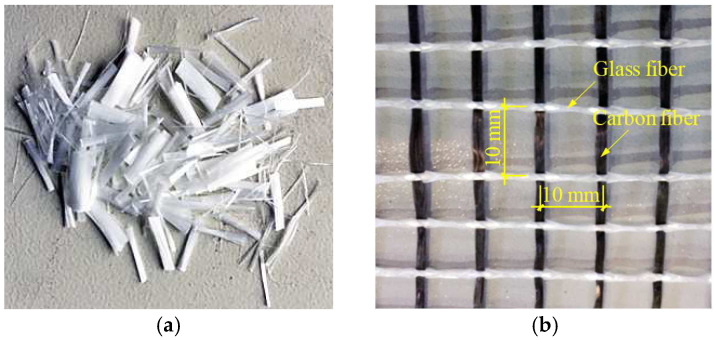
The images of (**a**) short-cut PVA fibers and (**b**) glass-carbon fabric woven net.

The matrix of the sprayed cement mortar is composed of Portland cement produced by Anhui Conch Cement Co., Ltd. (Wuhu, China); Class II fly ash, fine sand, and silica fume from Elkem International Trade (Shanghai, China) Co., Ltd. (Shanghai, China); and metakaolin, the thixotropic agent from BYK-Chemie GmbH, and superplasticizer from BASF Group; as shown in Table 1, and its water/binder ratio is 0.25. The measured compressive strength of the sprayed cement mortar prepared was 57.3 MPa. In specimen preparation, two types of fibers are used for reinforcement. One is short-cut polyvinyl alcohol fiber (PVA) supplied by Kuraray Co. Ltd. (Tokyo, Japan), with a length of 8 mm, which is randomly dispersed in the cement mortar matrix with a volume fraction of 2%. The other is a two-dimensional fabric woven net composed of alkali-resistant E-glass fiber yarn and carbon fiber yarn (T300), with a mesh size of 10 mm × 10 mm. In addition, the amount of superplasticizer was increased to 5 kg/m^3^ to ensure that the slump extension of PVA short-cut fibers reinforced cement mortar meets the spraying requirements of 700 mm. Table 2 and Table 3 [43] list the mechanical properties of PVA fiber and carbon fiber yarn used here, respectively. Figure 1 shows the images of 8 mm long short-cut PVA fibers and glass-carbon fabric woven net.

### 2.2. Specimens and Preparation

**Figure 2:**
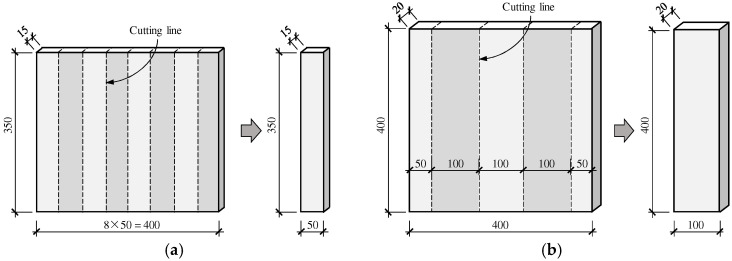
Schematics of (**a**) tensile specimen and (**b**) bending specimen cutting from an entire thin plate.

**Figure 3:**
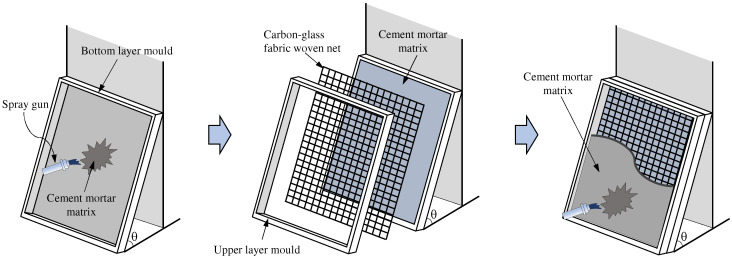
Preparation schematic of FRCM series thin plates with the fabric woven net.

Table 4 indicates the names, dimensions, and types of fibers used in FRCM specimens, as well as the number of specimens, in which SP, CN, and PN, respectively, represent the use of the non-directional short-cut PVA fibers, one layer of directional carbon-glass fabric woven net, and the short-cut PVA fibers mixed with one layer of directional carbon-glass fabric woven net. T and B represent the direct tensile test and four-point bending test, respectively. The four-point bending specimen with a size of 20 mm × 100 mm × 400 mm was cut from a thin plate with a size of 20 mm × 400 mm × 400 mm, as shown in Figure 2a. The direct tensile specimen with the size of 15 mm × 50 mm × 350 mm was cut from the thin plate with the size of 15 mm × 350 mm × 400 mm, as shown in Figure 2b. The cement mortar matrix of FRCM series specimens was prepared by spraying, as shown in Figure 3, in which the angle of the wooden mold was 80 degrees close to the actual construction conditions. The preparation of the three types of specimens was different in that: the FRCM-SP specimens were first mixed with the short-cut PVA fibers in the cement mortar matrix, and then the PVA fiber reinforced cement mortar was sprayed in the wood mold at one time; for the FRCM-CN specimen, a layer of 7.5 mm (tensile specimen) or 10 mm (four-point bending specimen) cement mortar was first sprayed in the bottom mold, then a carbon-glass fiber woven net without sizing was laid and pressed on the upper wood mold, and finally, a layer of 7 mm (tensile specimen) or 10 mm (four-point bending specimen) cement mortar was sprayed and leveled. The preparation method of FRCM-PN was the same as that of the FRCM-CN specimen, except that the matrix material was replaced by the PVA fiber-reinforced cement mortar. The prepared thin plate specimen was placed in the wood mold for 24 h and moved to the standard curing room with a temperature of 20 °C and humidity of 95% for 7 days. Then, the specimen was molded and cut into corresponding specimens and continued curing until 28 days later for the test.

### 2.3. Testing Method

**Figure 4:**
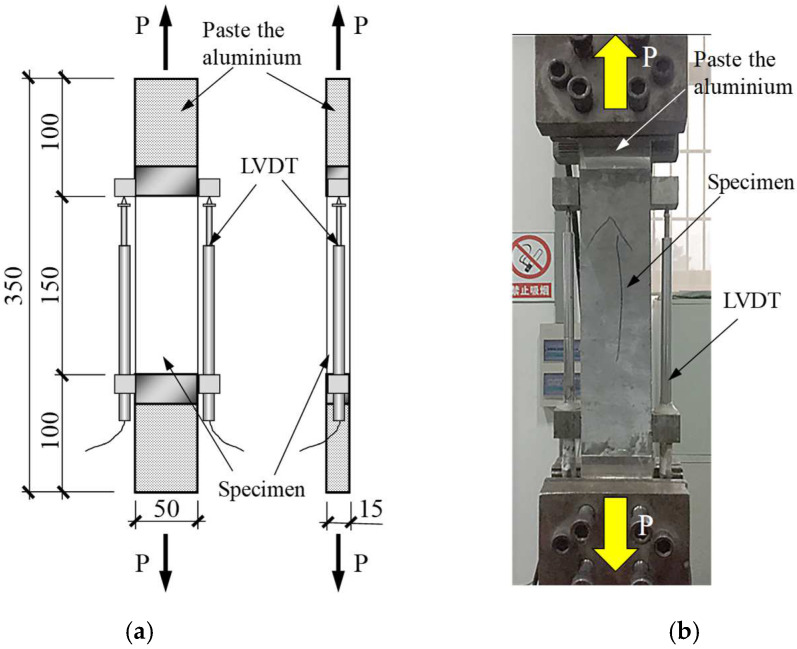
Direct tensile test device and specimen. (**a**) Dimensions and LVDTs arrangement; (**b**) photo of the test. Note: the unit for the numbers is mm.

**Figure 5:**
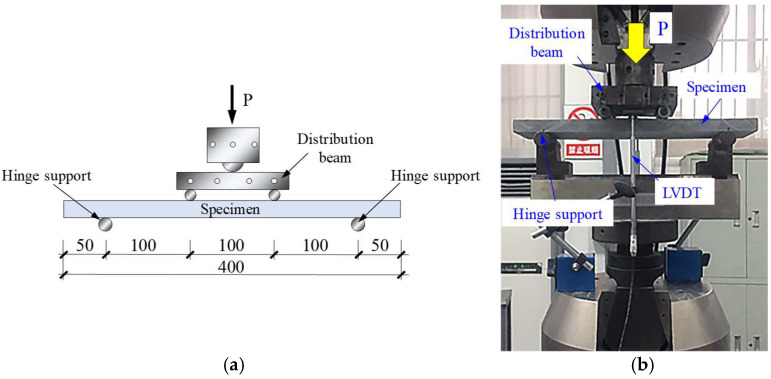
Four-point bending test device and specimen. (**a**) dimensions and loading device arrangement; (**b**) photo of the test. Note: the unit for the numbers is mm.

The loading devices, specimen dimension, and linear variable differential transformer (LVDT) measuring points of the direct tensile test and four-point bending test are shown in Figure 4 and Figure 5, respectively, all of which are loaded by a 25 t Instron fatigue testing machine, Instron, Norwood, MA, USA. Because the specimens’ dimensions were non-standard, the tensile and bending tests were designed according to the methods of Refs. [38,44], respectively. As shown in Figure 4a, the aluminum sheets were pasted at both ends of the direct tensile specimen with a length of 100 mm to avoid stress-concentrated failure caused by the clamping device on both ends of the specimen. Two LVDTs were arranged symmetrically on both sides of the tensile specimen to test the tensile deformation within 150 mm of the middle length of the specimen. Similarly, two LVDTs were symmetrically arranged in the mid-span on both sides of the bending specimen to test the deflection deformation at the top of the mid-span of the thin plate, as shown in Figure 5b.

Both the direct tensile test and the bending test adopted the displacement-controlled loading mode, and the loading rates were 0.2 mm/min and 0.5 mm/min, respectively. The load value in the test process was read and recorded by the load sensor of the testing machine.

## 3. Experimental Results and Discussion

The specimens’ crack propagation and failure mode were observed and analyzed by direct tensile and four-point bending tests. We measured the developed length of cracks at different loading stages with a common ruler and the width of the main cracks with the concrete crack width observation instrument. The tensile and bending toughness indexes of different specimens were analyzed based on the obtained load and displacement data.

### 3.1. Crack Propagation Analysis and Failure Mode

**Figure 6:**
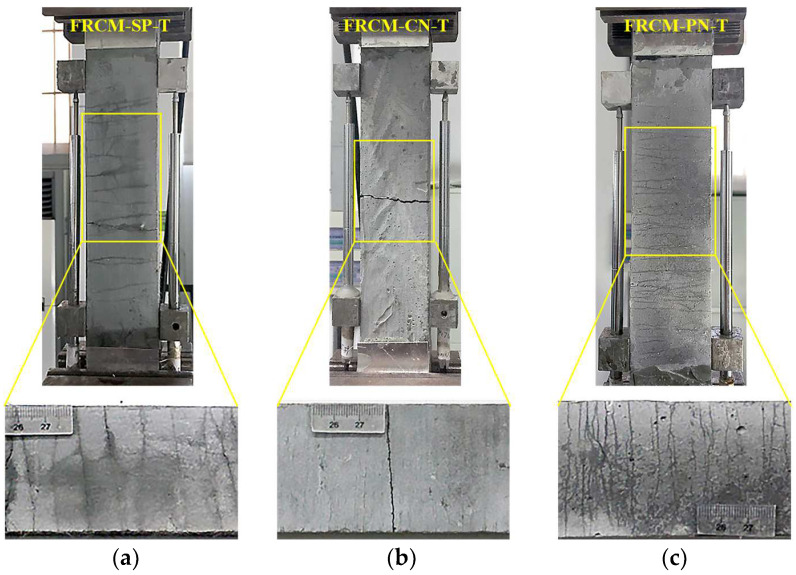
Crack images of tensile specimens. (**a**) FRCM-SP-T; (**b**) FRCM-CN-T; (**c**) FRCM-PN-T.

**Figure 7:**
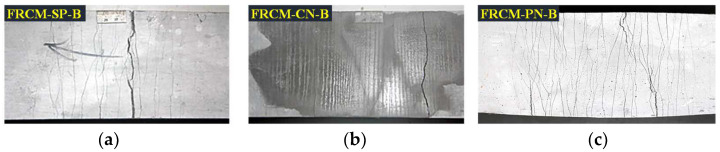
Crack images of bending specimens. (**a**) FRCM-SP-B; (**b**) FRCM-CN-B; (**c**) FRCM-PN-B.

The crack distributions of the three types of specimens after direct tensile and four-point bending tests are shown in Figure 6 and Figure 7, respectively. The maximum crack widths measured for direct tensile tests loaded to 1% and 2% tensile strain are shown in Table 5. The width, number, spacing of the cracks, and final failure mode observed in the bending tests when the mid-span deflection reached 5 mm, 10 mm, and 15 mm, respectively, are shown in Table 6. As can be seen from these figures, the FRCM-CT specimens showed a brittle damage pattern with a single primary crack under both direct tensile and bending conditions, which may be due to the poor interfacial bonding performance between the cement mortar matrix and the oriented fiber yarns, resulting in the fiber yarns pulling out from the matrix soon after the initial cracking occurred and not transferring the interfacial bond stress well. Although the presence of compressive stress at the interface between the fabric woven net and the cement mortar matrix in the four-point bending test improved the interfacial bonding performance and enhanced the toughness of the sprayed cement mortar to some extent, only three to five fine cracks in addition to the main crack were observed.

In order to meet the demand for sprayed fiber-reinforced cement mortar, the length of non-directional short-cut PVA fibers was changed to 8 mm compared with that used in conventional cast ECC. FRCM-SP differed from ECC in terms of matrix components and raw material ratio but still exhibited a ductile damage pattern with multiple cracking in direct tensile and four-point bending tests. The crack width was 50–80 μm and 70–100 μm at 1% and 2% tensile strain, respectively, and the crack spacing remained between 4 and 9 mm.

When the sprayed cement mortar was toughened by the combination of directional fabric woven net and non-directional short-cut PVA fibers, the FRCM-PN specimens exhibited a ductile damage mode with multiple cracks cracking in both direct tensile and four-point bending tests. As shown in Table 5, the number of cracks produced under direct tensile loading and bending loading reached 59 ± 3 and 81 ± 3, respectively, which was almost twice as many as that of the FRCM-SP specimens. Moreover, the cracks were so fine that they were hardly detectable by the naked eye in the dry state and could only be seen after wiping the specimen surface with anhydrous alcohol. The crack width was 20–30 μm and 40–60 μm at 1% and 2% tensile strain, respectively, and the crack spacing remained between 2 and 5 mm. In addition, the crack widths were 20–30 μm, 40–50 μm, and 50–70 μm at bending deflections of 5 mm, 10 mm, and 15 mm, respectively, while the crack spacing also remained between 2 and 5 mm.

**Figure 8:**
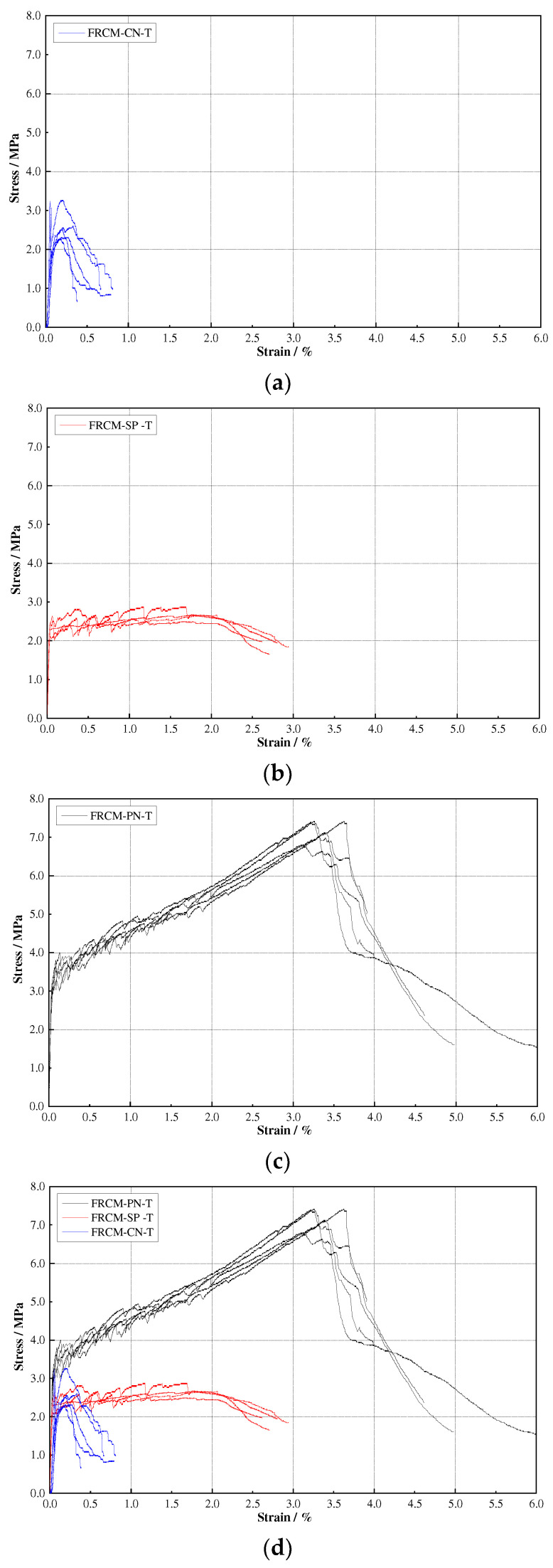
Tensile stress-strain curves for (**a**) FRCM-CN-T, (**b**) FRCM-SP-T, and (**c**) FRCM-PN-T, and (**d**) comparison of three types of specimens.

**Figure 9:**
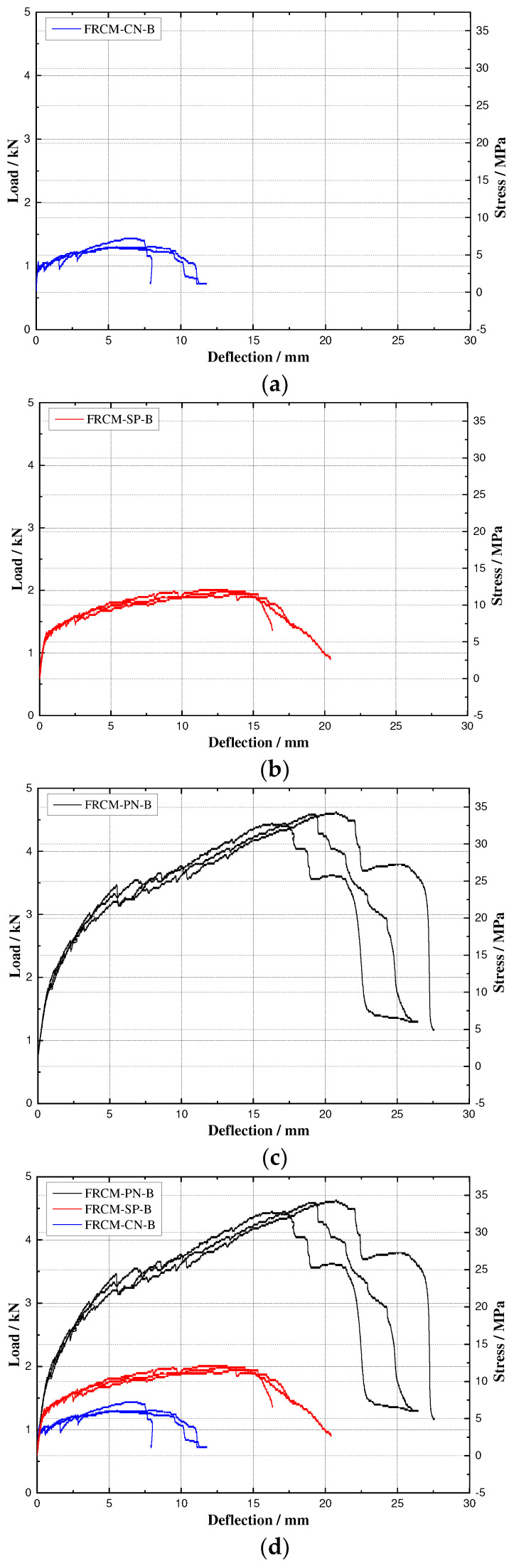
Flexural load/equivalent stress-deflection curves for (**a**) FRCM-CN-T, (**b**) FRCM-SP-T, (**c**) and FRCM-PN-T, and (**d**) comparison of three types of specimens.

Combining the crack development and stress-strain curves (see Figure 8 and Figure 9), we can divide the whole crack development process of all specimens into three stages, i.e., the elastic stage before the initial crack appears, the elastic-plastic stage when multi-fine cracks crack, and the damage stage when the main crack develops. However, there are differences between the three types of specimens. For the FRCM-CT specimen, because the fabric woven net was not treated with impregnation, the interfacial bonding between fiber bundles and the cement matrix is not good. After the first crack appears, the stress of the cracked matrix is rapidly transferred to the fiber bundle, and the interfacial bonding stress cannot balance the sudden increase in tensile stress of the fiber bundle, resulting in the fiber bundle slowly sliding out of the matrix and debonding, then the crack gradually opens wider, and the crack length and depth gradually penetrate the specimen. Although two or three accompanying fine cracks appear next to the main crack, both the direct tensile and bending tests exhibit a brittle damage mode. For the FRCM-SP specimen, due to the strong cross-sectional bonding of short-cut PVA fibers and cement matrix, after the first initial crack appeared, the uniformly distributed short-cut PVA fibers played a bridging role at both sides of the crack, so that the stress in the crack cross-section was uniformly transferred to the non-cracking zone. With the increase in load, the cracks did not show obvious widening under the bridging effect of fibers, while fine cracks gradually appeared in the other uncracked zone, which showed stress-hardening stages in the stress-strain curve until the fibers were finally pulled out from the cement matrix or the fibers were pulled off, showing a ductile damage mode. For the FRCM-PN, the composite is mixed with short-cut PVA fibers and fabric woven net, so the short-cut PVA fibers make up for the defect of insufficient bonding between the fiber bundle and the matrix, which makes the crack cracking mode of this series of specimens closer to that of FRCM-SP, which also shows the ductile damage mode. Moreover, the tensile strength and the bending strength were greatly increased.

The above experimental phenomena show that the incorporation of non-directional short-cut PVA fibers enhances the interfacial bonding performance between the fiber bundle and the sprayed cement mortar matrix, which not only makes the sprayed FRCM exhibit better crack control and ductility but also improves its mechanical properties.

### 3.2. Tensile and Flexural Experiment Results

The direct tensile stress-strain curves of FRCM-SP-T, FRCM-CN-T, and FRCM-PN-T are shown in Figure 8, and the bending load/stress-deflection curves of FRCM-SP-B, FRCM-CN-B, and FRCM-PN-B are shown in Figure 9. In the direct tensile test, the stress and strain of the thin-plate specimens are calculated using the Formulas (1) and (2), while in the four-point bending test, the equivalent flexural stress is obtained by Formula (3), and the deflection of the thin-plate is the deflection of the mid-span.

(1) $$\sigma_{t}=P/(t\times b),$$

(2) $$\varepsilon_{t}=\Delta /d_{0},$$

(3) $$\sigma_{f}=Pl_{0}/bt^{2},$$

where $\sigma_{t}$ and $\sigma_{f}$ are the tensile stress and equivalent flexural stress, respectively, and *P* is the tensile load or flexural load recorded by the load sensor of the testing machines. $\varepsilon_{t}$ refers to tensile strain, and Δ indicates the extension of tensile specimens along the gauge length *d*_0_, which is 150 mm; *l*_0_ is the span length of bending test specimens; *t* and *b* are the thickness and width of the thin plate, respectively.

Table 7 shows the strength and strain values corresponding to the initial cracking and limit state in the direct tensile test and four-point bending test, where the initial cracking strength and strain are defined as the stress and strain corresponding to the point at which the linear stage of the curve changes to the nonlinear stage, as shown in Figure 8 and Figure 9.

When the FRCM-SP specimen is subjected to tensile stress exceeding the cracking stress of the cement mortar matrix, cracks will first be generated at the defective part of the specimen, but the short-cut PVA fibers with uniform disorderly distribution will tightly pull the matrix on both sides of the cracks through the bridging action, thus controlling the further expansion of the cracks, and transferring the tensile stress at the cracks to the uncracked matrix on both sides through the bonding force between the fiber yarns and the cement mortar matrix. This aforementioned cracking-crack-arresting process occurred repeatedly during the loading process of direct tension and four-point bending tests, causing the specimens to exhibit multiple cracks cracking and strain hardening, and also accounting for the small up-and-down jitter in the stress-strain curve. The average strength of the FRCM-SP-T specimens was 2.62 MPa, which was 16.96% higher than the average initial cracking strength of 2.24 MPa. The equivalent ultimate average bending strength was 11.92 MPa, which was 70.77% higher than the bending initial cracking strength of 6.98 MPa.

For FRCM-CN-T specimens subjected to direct tensile loads, the first crack appears when the tensile stress in the section is greater than the matrix cracking stress and will rapidly develop into a penetration crack. At this point, the tensile load will suddenly drop to less than 50% of the initial cracking load. Thereafter, the tensile load rises again due to the directional reinforcement of the carbon fiber bundle, which effectively transfers the tensile stress at the cracked interface. Since the outer filaments of the carbon fiber bundle can be completely infiltrated by the matrix and form good anchorage with the cement mortar matrix, when the matrix cracks, the interfacial tensile stresses are transferred to the outer filaments of the carbon fiber bundle and the outer filaments bear the higher tensile stresses, while the inner filaments transfer some of the tensile stresses through inter filament friction. With the increase in tensile load, these carbon fiber filaments bearing high stresses break or peel off from the matrix, resulting in the gradual failure of the anchorage between the carbon fiber bundle and the cement mortar matrix. This phenomenon is reflected in the stress-strain curve for the hardened stage as well as the softened stage after the ultimate stress. The average ultimate tensile strength of the FRCM-CN-T specimen is 3.42 MPa.

For the FRCM-CN-B specimens subjected to bending loads, when the specimens were initially cracked, the width and depth of the first crack grew rapidly with increasing load and gradually extended to the interface of the fiber woven net. Subsequently, the bending stress was transferred to the uncracked matrix on both sides of the crack through the bridging action of the carbon fiber bundles, and a second crack and multiple other cracks gradually developed. All FRCM-CN-B specimens showed no further increase in the number of cracks after the appearance of 3–5 cracks, but the width kept increasing, i.e., the carbon fiber bundles of the fiber woven net and the matrix could not deform synergistically, and a slip phenomenon was generated. The bending load-deformation curve showed a descending stage until the bending specimen was severely damaged and failed. The average equivalent bending stress of the FRCM-CN-B specimen was 6.48 MPa, which was 69.2% higher than its initial bending cracking stress of 3.83 MPa, but it might still be small for engineering applications.

From the above comparison, we find that the ultimate tensile strength of FRCM-CN is higher than that of FRCM-SP, but the ultimate bending strength is the opposite. In the direct tensile test, we pasted aluminum sheets to both ends of the specimen and clamped the ends with a fixture for the tensile test. Due to the clamping force applied to the specimen ends, the FRCM-CN series actually has the effect of pulling fiber bundles directly by the clamping force after cracks appear. In contrast, FRCM-SP controls crack development through the bridging action of short-cut PVA fibers, and its stress transfer efficiency is lower than that of directly stretched fiber bundles. Therefore, the tensile strength of FRCM-CN is higher than that of FRCM-SP. However, in the bending test, there is no restraint at the end of the specimen, so the stress transfer is not as strong as that of the short-cut PVA fibers due to the weak interfacial action between the fiber bundle and the cement matrix; this shows that the bending strength of FRCM-CN is smaller than that of FRCM-SP.

FRCM-PN specimens sustained the properties of strain-hardening and multiple-fine-cracking, which FRCM-SP specimens are characterized by. Moreover, the tensile and flexural performance is far better than those of FRCM-SP or FRCM-CN due to the cooperative reinforcement of short-cut fibers and carbon fabric woven net. FRCM-PN possesses an average initial cracking strength and ultimate strength of 3.76 MPa and 7.22 MPa, respectively, during direct tensile tests and an average initial cracking strength and ultimate strength of 9.49 MPa and 33.67 MPa, respectively, during bending tests. The ratio of the average ultimate strength to initial cracking strength of tensile specimens has an increase of 92.0%, while that of bending specimens is 254.8%. The average ultimate tensile strength of FRCM-PN is 175.6% and 111.1% higher than that of FRCM-SP and FRCM-CN, respectively, and FRCM-PN possesses an ultimate tensile strain of 65.3% and 1291.7% higher than FRCM-SP and FRCM-CN, respectively. The average equivalent bending ultimate strength of FRCM-PN is 182.5% and 419.6% higher than that of FRCM-SP and FRCM-CN, respectively, while the ultimate flexural deformation of FRCM-PN is 21.2% and 129.4% better than that of FRCM-SP and FRCM-CN, respectively.

### 3.3. Tensile and Flexural Toughness

**Figure 10:**
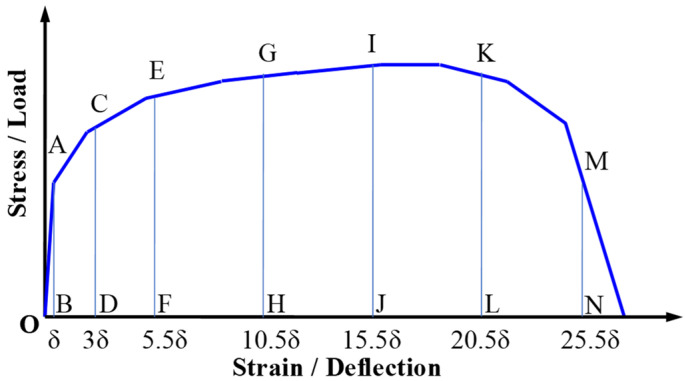
The schematic diagram for calculating toughness indexes and residual strength factors. Note: the letters B, D, F, etc. on the horizontal coordinate indicate the points corresponding to δ, 3δ, 5.5δ, etc., and the letters A, C, E, etc. on the curve indicate the points corresponding to the stress/load values when the strain/deflection is 3δ, 5.5δ, etc., respectively.

A schematic diagram for calculating the toughness indexes and residual strength factors is shown in Figure 10. A toughness index *I*_T_ is defined to estimate the toughness and energy absorption capacity of materials according to the ASTM C 1018 standard [45], which is calculated by Formula (4). Shwan H. Said et al. [46] carried out some experiments to investigate the flexural toughness of ECC thin-plate, and *I*_T_ was calculated as ASTM C 1018. Moreover, another index termed residual strength factor *R*_S, T_ was also investigated in [46], and *R*_S_, _T_ was calculated by Formula (5).

(4) $$I_{T}=\frac{A_{n\delta }}{A_{\delta }},$$

(5) $$R_{S,\;T}=N\left(I_{T}-I_{S}\right),$$

(6) $$N=100/\left(T-S\right)$$

where *I*_s_ is also the toughness index and denotes any of the toughness stands for when S is less than T; T or S takes the value 2n − 1, and n is equal to 1, 3, 5.5, 10.5, etc.; *A*_nδ_ is the area under the flexural load-deflection or tensile-strain curve up to a limited deflection or strain value; *A*_δ_ is the area under the flexural load-deflection or tensile-strain curve up to the initial cracking deflection or strain value; δ is a flexural initial cracking deflection or tensile initial cracking strain.

These two indexes and factors are also considered in this paper, and they are used to evaluate the flexural toughness of FRCM-PN and to compare it with the flexural toughness of two other types of specimens. Some certain fiber-reinforced cementitious composites can be characterized by deformation hardening property during flexural test progress, but tension-softening behavior was observed in tensile tests [47], so it is actually the same as FRCM-CN in this paper. Furthermore, the tensile test is usually used as a kind of material testing method while the flexural test is often seen as a kind of structure testing method. Therefore, it is more reasonable to judge by direct tensile tests whether a certain material is a strain-hardening material or not [48]. Therefore, the tensile toughness of FRCM-PN, FRCM-SP, and FRCM-CN was also calculated and discussed similarly based on Formulas (4) and (5).

For brittle materials, the toughness index and residual strength factor are equal to 1 and 0, respectively. As for elastic-perfectly plastic materials, the toughness factor is equal to the subscript T of toughness index *I*_T_, and the residual strength factor *R*_S, T_ is equal to 100 according to the formulas. So, if a certain kind of material is characterized by strain-hardening property, its toughness index *I*_T_ should be larger than the corresponding subscript T, and the residual strength factors *R*_S, T_ should be larger than 100. The larger the ratio of *I*_T_ to T is, or the larger value of *R*_S_, _T_/100 is, the higher the toughness will be; the same is true for the energy absorption capacity.

**Figure 11:**
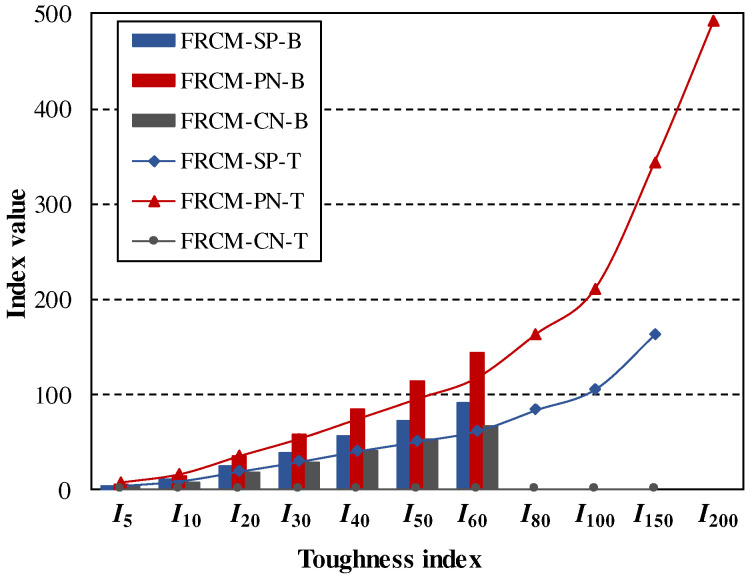
Toughness indexes during tensile and bending tests.

The flexural and tensile toughness indexes of FRCM-SP, FRCM-PN, and FRCM-CT were calculated and are listed in Table 8. All the toughness indexes of FRCM-SP and FRCM-PN are larger than their corresponding subscripts T of *I*_T_, which apparently proves that both FRCM-SP and FRCM-PN can be characterized by strain hardening or deformation hardening. This is not the same case for FRCM-CN, whose tensile toughness index is equal to 1, and some toughness indexes are smaller than the subscript of *I*_T_. It is worth noticing that the flexural toughness index generally sustains a larger value of *I*_T_ than that of direct tensile one, as shown in Figure 11, which shows that the flexural performance of a certain material may be better than its tensile performance. So, overestimation of the performance of a material may occur when only the flexural performance is taken into consideration, and it should be more conservative but safer when the tensile performance is applied to estimating the material property if tensile tests could be conducted. Moreover, the values of every *I*_T_ of FRCM-PN are much larger than those of FRCM-SP and FRCM-CN, which shows that FRCM-PN possesses a much higher toughness and greater capability of energy consumption than FRCM-SP and FRCM-CN.

**Figure 12:**
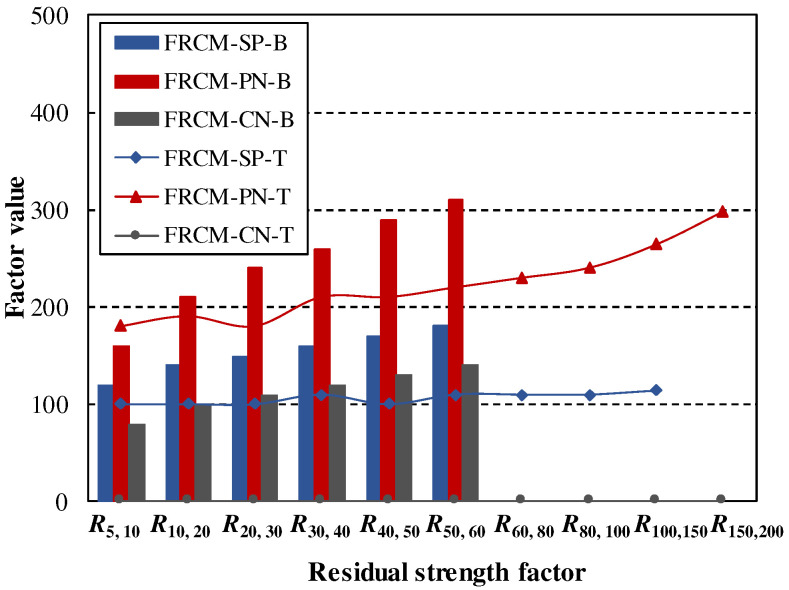
Residual strength factors during tensile and bending tests.

Table 9 lists the flexural and tensile residual strength factors of FRCM-SP, FRCM-PN, and FRCM-CN, whose comparison relation is shown in Figure 12. FRCM-CT possess tensile residual strength factors of 0 but much larger flexural residual strength factors even exceeding 100, while the tensile *R*_S_, _T_ values of FRCM-SP are slightly larger than 100 and the flexural ones are in the range of 120~180. FRCM-PN possess residual strength factors much larger than FRCM-SP and FRCM-CT for both tensile tests and flexural tests, which also shows a much better capability of toughness and energy absorption. It is also indicated that the residual strength factors of testing specimens are larger than their tensile ones with the same fiber-reinforced same way of toughness indexes.

In addition, the microscopic experimental studies by Shen et al. [44,49] show that short-cut PVA fibers play a bridging role at the stage of tiny cracks in the cement matrix. Moreover, for the fabric woven net without impregnation treatment, it often has defects such as pores at the interface with the cement matrix, and the interfacial bonding performance is not good, while the short-cut PVA fibers make up for the defects and also play a bridging role between the matrix and the fiber bundles, improving the interfacial bonding performance and stiffness, flexural strength and deflection of the FRCM thin plates. Therefore, these microscopic mechanisms also effectively explain the better performance of the thin plates in terms of strength, toughness, and multiple cracking failure modes after composite doping of short-cut PVA fibers and fiber woven net in this study.

## 4. Conclusions

Based on the spraying construction process, FRCM thin plates were prepared using sprayed cement mortar as the matrix and reinforced with non-directional short-cut PVA fibers and/or a directional fabric woven net, and uniaxial tensile and four-point bending tests were performed. The flexural/tensile toughness indexes and residual strength factors of each specimen were calculated and discussed according to the ASTM C 1018 standard. The conclusions are summarized as follows:

(1) In the uniaxial tensile test of FRCM-CN series thin plates, specimens show brittle and single-crack failure behavior, which indicates that the fiber woven net and cement mortar matrix do not show good bonding or a cooperative working effect, resulting in the tensile properties of the fiber woven net not being effectively utilized. However, in the four-point bending tests, the specimens show good flexural toughness, which indicates that the fiber woven net and the cement mortar matrix have a good cooperative working effect under the composite tensile, shear, and compressive stress of the section. Therefore, only considering uniaxial tensile mechanical indexes to evaluate the mechanical properties of such materials in structural design may be conservative, but relatively safe.
(2) The toughness indexes of FRCM-SP and FRCM-PN were greater than the corresponding subscripts, and the residual strength factors were greater than 100, indicating that both of them are strain-hardening materials. Moreover, the toughness index and residual strength factor of FRCM-PN are greater than that of FRCM-SP. The above phenomenon indicates that a certain amount of PVA short-cut fiber makes the cement mortar matrix form strain-hardening material, which further improves the strength and toughness under the co-action with the fabric woven net.
(3) In the failure stage, the cracks observed in FRCM-PN are finer and denser than those observed in FRCM-SP and FRCM-CN, which proves that adding short-cut fibers into the sprayed cement mortar matrix can effectively enhance the cooperative working performance of the mortar matrix and the fiber yarn and improve the crack control ability of FRCM-CN.
(4) In this study, there are limitations in the selection of test parameters and the constraint of direct tensile tests, and the interfacial bonding mechanism between the matrix and fiber bundles is not investigated in more depth. Therefore, in the future, we will conduct more in-depth studies on the multilayer fabric woven net reinforced cement mortar, the constraint treatment of the tests, the interfacial bonding mechanism between matrix and fabric bundles, and its relationship with the macroscopic mechanical properties, and will provide basic data for the engineering application and promotion of FRCM.

## Figures and Tables

**Table 1:** The proportion of sprayed cement mortar (kg/m^3^).

Cement	Fly Ash	Silica Fume	Fine Sand	Metakaolin	Thixotropic Agent	Superplasticizer
452	198	45	1400	50	1.0	3.5

**Table 2:** Properties of PVA fiber [43].

Tensile Strength (MPa)	Diameter (μm)	Fiber Length (mm)	Young’s Modulus (GPa)	Elongation (%)
1600	39	8	40	6

**Table 3:** Properties of carbon yarns of fabric woven net.

Tensile Strength (MPa)	Tex (g/km)	Density (g/cm^3^)	Theoretical Area (mm^2^)	Young’s Modulus (GPa)	Elongation (%)
4900	800	1.82	0.44	230	1.35

**Table 4:** Specimen index and main information.

Specimen	Test Method	Dimension (mm)	Number	Fiber Type
FRCM-SP-T	Tensile	15 × 50 × 350	6	Short-cut PVA
FRCM-SP-B	Bending	20 × 100 × 400	3	Short-cut PVA
FRCM-CN-T	Tensile	15 × 50 × 350	6	Glass-carbon woven net
FRCM-CN-B	Bending	20 × 100 × 400	3	Glass-carbon woven net
FRCM-PN-T	Tensile	15 × 50 × 350	6	PVA + woven net
FRCM-PN-B	Bending	20 × 100 × 400	3	PVA + woven net

**Table 5:** Crack propagation situation during the tensile test.

Specimens	FRCM-SP	FRCM-PN	FRCM-CT
w-1%	50~80 μm	20~30 μm	failure
w-2%	70~100 μm	40~60 μm	failure
Crack number	31 ± 3	59 ± 3	1
Crack spacing	4~9 mm	2~5 mm	-
Failure mode	ductile mode	ductile mode	brittle mode

**Table 6:** Crack propagation situation during the bending test.

Specimens	FRCM-SP	FRCM-PN	FRCM-CT
w-5	40~60 μm	20~30 μm	>0.5 mm
w-10	60~80 μm	40~50 μm	>1.0 mm
w-15	80~100 μm	50~70 μm	>2.0 mm
Crack number	48 ± 3	81 ± 3	3~5
Crack spacing	3~8 mm	2~5 mm	>10 mm
Failure mode	ductile mode	ductile mode	brittle mode

**Table 7:** The results of tensile testing and flexural testing.

Specimen	Tensile Stiffness (GPa)	Initial Cracking Strength (MPa)	Initial Cracking Strain (%)	Ultimate Strength (MPa)	Ultimate Strain (%)
FRCM-SP-T	7.85	2.24 (0.059)	0.026	2.62 (0.33)	2.02
FRCM-CN-T	5.92	3.14 (0.216)	0.053	3.42 (0.151)	0.24
FRCM-PN-T	14.5	3.76 (0.109)	0.026	7.22 (0.208)	3.34
**Specimen**	**Bending Stiffness (GPa)**	**Initial Cracking Strength (MPa)**	**Initial Cracking Strain (%)**	**Ultimate Strength (MPa)**	**Ultimate Deflection (mm)**
FRCM-SP-B	15.86	6.98 (0.238)	0.044	11.92 (0.132)	15.82
FRCM-CN-B	23.93	3.83 (0.124)	0.016	6.48 (0.17)	8.36
FRCM-PN-B	16.08	9.49 (0.134)	0.059	33.67 (0.391)	19.18

Note: the values in parentheses represent the standard deviation.

**Table 8:** Toughness indexes during bending tests and tensile tests.

Flexural	*I*_5_	*I*_10_	*I*_20_	*I*_30_	*I*_40_	*I*_50_	*I*_60_	*I*_80_	*I*_100_	*I*_150_	*I*_200_	*I*_U_
FRCM-SP	5	11	25	40	56	73	91	-	-	-	-	94
FRCM-PN	6	14	35	59	85	114	145	-	-	-	-	139
FRCM-CN	4	8	18	29	41	54	68	-	-	-	-	131
**Tensile**	***I*_5_**	***I*_10_**	***I*_20_**	***I*_30_**	***I*_40_**	***I*_50_**	***I*_60_**	***I*_80_**	***I*_100_**	***I*_150_**	***I*_200_**	***I*_U_**
FRCM-SP	5	10	20	30	41	51	62	84	106	163	-	166
FRCM-PN	7	16	35	53	74	95	117	163	211	343	492	656
FRCM-CN	1	1	1	1	1	1	1	1	1	1	1	1

**Table 9:** Residual strength factors during bending tests and tensile tests.

Flexural	*R*_5,10_	*R*_10,20_	*R*_20,30_	*R*_30,40_	*R*_40,50_	*R*_50,60_	*R*_60,80_	*R*_80,100_	*R*_100,150_	*R*_150,200_
FRCM-SP	120	140	150	160	170	180	-	-	-	-
FRCM-PN	160	210	240	260	290	310	-	-	-	-
FRCM-CN	80	100	110	120	130	140	-	-	-	-
**Tensile**	***R*_5,10_**	***R*_10,20_**	***R*_20,30_**	***R*_30,40_**	***R*_40,50_**	***R*_50,60_**	***R*_60,80_**	***R*_80,100_**	***R*_100,150_**	***R*_150,200_**
FRCM-SP	100	100	100	110	100	110	110	110	114	-
FRCM-PN	180	190	180	210	210	220	230	240	264	298
FRCM-CN	0	0	0	0	0	0	0	0	0	0

## Data Availability

The raw/processed data required to reproduce these findings cannot be shared at this time as the data also form part of an ongoing study. The datasets generated during and/or analyzed during the current study are available from the corresponding author upon reasonable request.
